# Supplementary material for: Genome-Wide Gene Expression Analysis Reveals Unique Genes Signatures of Epithelial Reorganization in Primary Airway Epithelium Induced by Type-I, -II and -III Interferons
Source: Biosensors (Basel). 2022 Oct 26;12(11):929. doi: 10.3390/bios12110929 (PMC9688329; doi:10.3390/bios12110929)

biosensors

MDPI

Supplementary Materials

Supplement Figure 1: Workflow of Air liquid interface culture and Microarray

Primary human bronchial epithelial cells of six genetically independent donors were first grown submerged and then transferred to polyester membrane. After fully diferentiation, cells were harvested for microarray analysis of hybridized RNA.

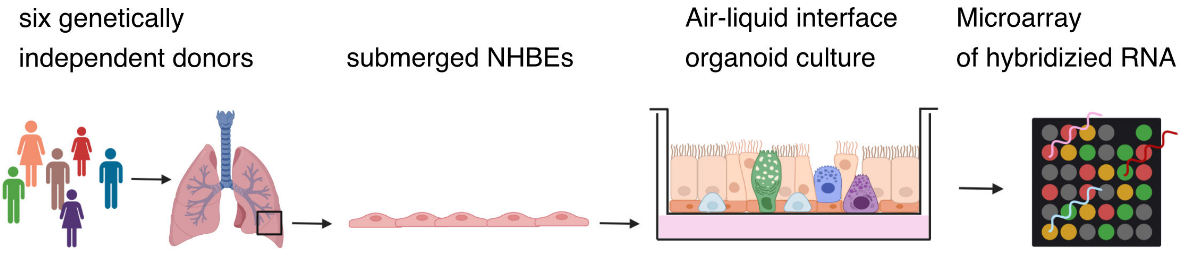

Supplement Figure 2: Anti-inflammatory genes induced by IFNα, type-II and type-III IFNs

A) Heat map of induced anti-inflammatory genes by type-I IFNs (ttest IFNα-stimulated vs unstimulated). Duplicate gene names indicate the abundance of two or more transcripts of the same gene in the analysis and are marked with a cross. B) Heat map of induced anti-inflammatory genes by type-III IFNs (ttest IFNλ1-stimulated; IFNλ3-stimulated vs unstimulated) C) Heat map of induced anti-inflammatory genes by type-II IFNs (ttest IFNγ-stimulated vs unstimulated).

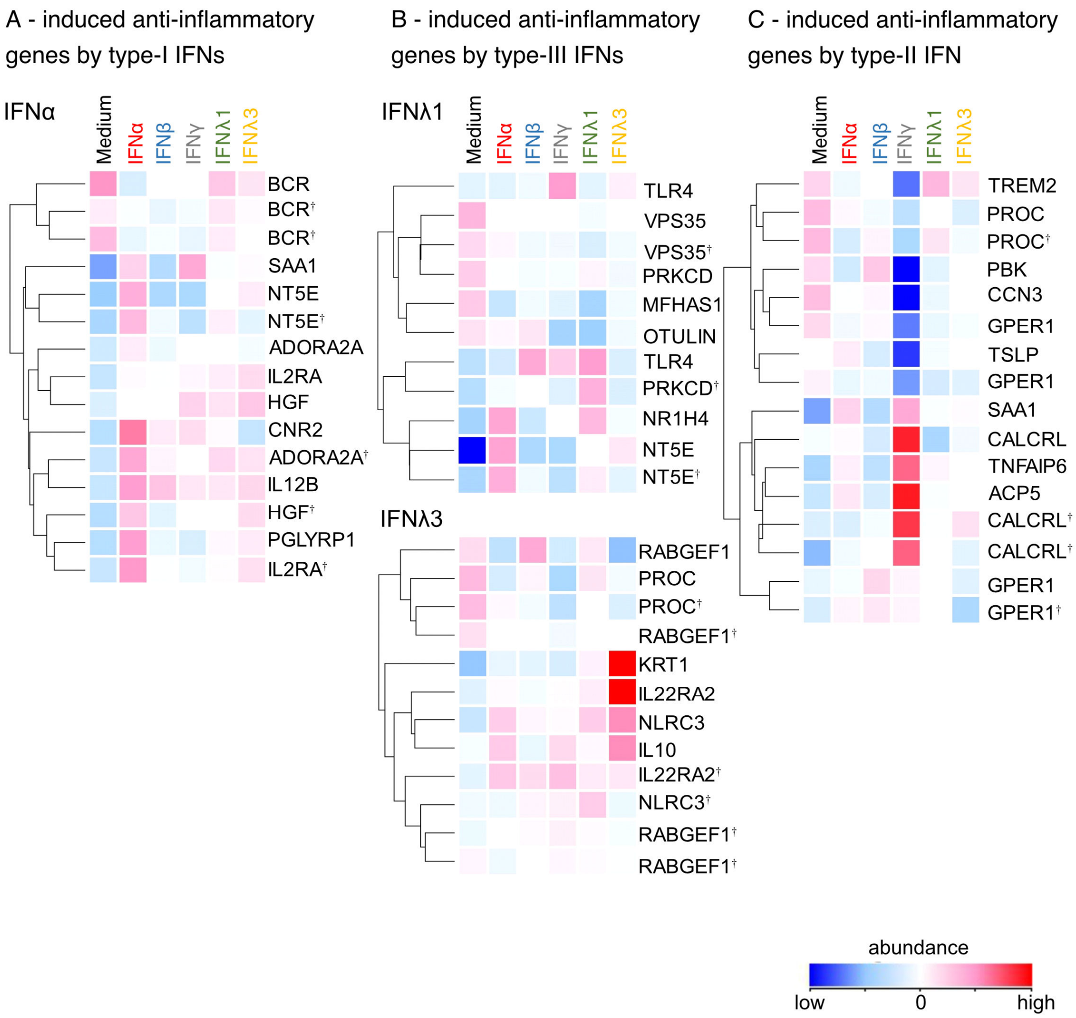
A - induced anti-inflammatory genes by type-I IFNs
B - induced anti-inflammatory genes by type-III IFNs
C - induced anti-inflammatory genes by type-II IFN
IFNα
IFNλ1
IFNλ3
Medium
IFNα
IFNβ
IFNγ
IFNλ1
IFNλ3
BCR
BCR†
BCR†
SAA1
NT5E
NT5E†
ADORA2A
IL2RA
HGF
CNR2
ADORA2A†
IL12B
HGF†
PGLYRP1
IL2RA†
TLR4
VPS35
VPS35†
PRKCD
MFHAS1
OTULIN
TLR4
PRKCD†
NR1H4
NT5E
NT5E†
RABGEF1
PROC
PROC†
RABGEF1†
KRT1
IL22RA2
NLRC3
IL10
IL22RA2†
NLRC3†
RABGEF1†
RABGEF1†
TREM2
PROC
PROC†
PBK
CCN3
GPER1
TSLP
GPER1
SAA1
CALCRL
TNFAIP6
ACP5
CALCRL†
CALCRL†
GPER1
GPER1†
abundance
low
0
high

Supplement Figure 1

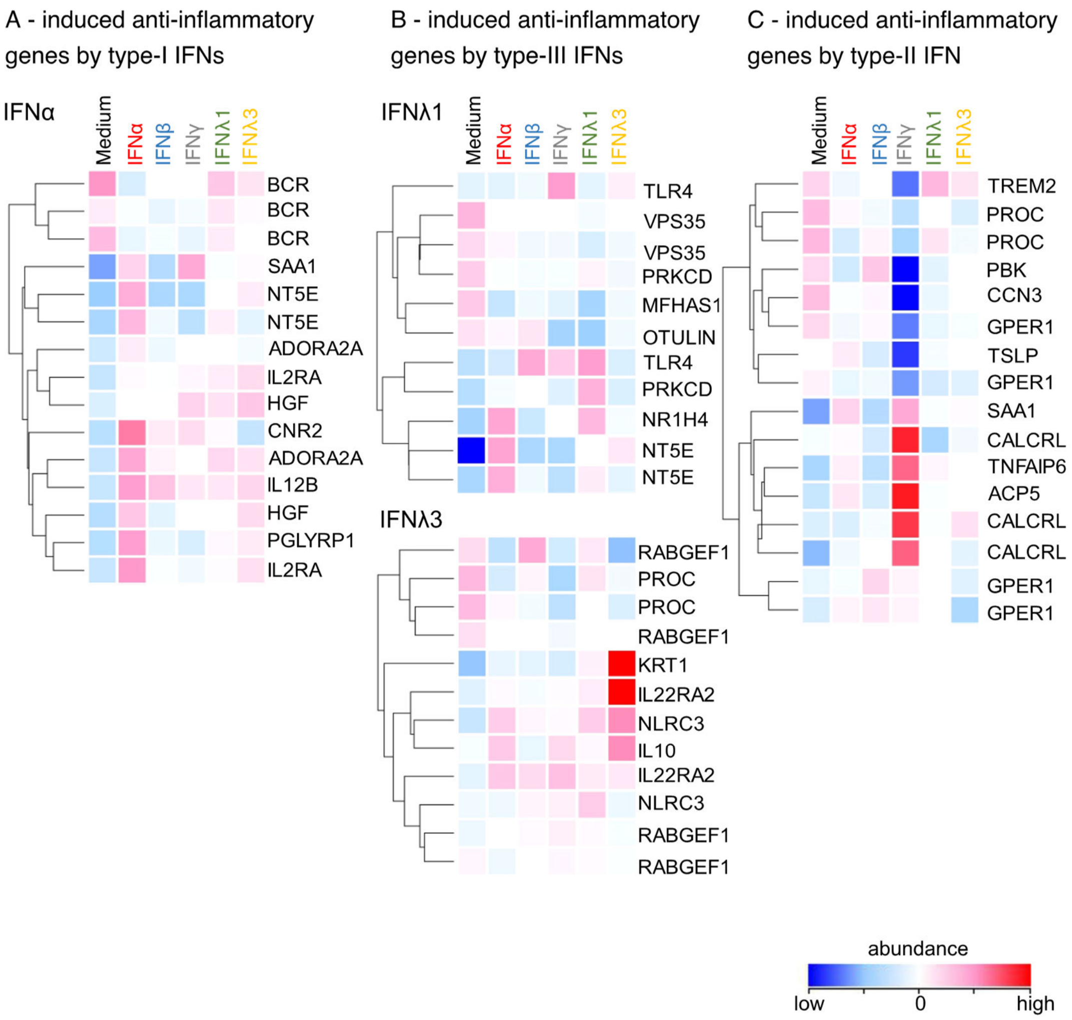

Supplement: Supplementary file 1 [file biosensors-12-00929-s001.zip › biosensors-1945460-supplementary.pdf]
